# Preliminary psychometric validation of patient-reported outcomes relevant to individuals with spinal muscular atrophy and their caregivers

**DOI:** 10.1186/s13023-025-03832-y

**Published:** 2025-06-04

**Authors:** Maria Grazia Cattinari, Samuel Ignacio Pascual-Pascual, Mencía de Lemus, Julita Medina, María Dumont, Pablo Rebollo, Juan Francisco Vázquez-Costa

**Affiliations:** 1 Fundacion Atrofia Muscular Espinal España (FundAME), Calle Nuria 93, 1ºC, Madrid, 28034 Spain; 2 Hospital Universitario La Paz, Universidad Autónoma de Madrid, Paseo de la Castellana 261, Madrid, 28046 Spain; 3 SMA Europe, Im Moos 4, 79112 Freiburg, Germany; 4 Committee of Advanced Therapies at the European Medicines Agency. Domenico, Scarlattilaan 6, Amsterdam, 1083 HS The Netherlands; 5 Hospital Sant Joan de Deu, Institut de Recerca San Joan de Deu, Pg. de Sant Joan de Déu, 2, Esplugues de Llobregat, Barcelona, 08950 Spain; 6 Iqvia, Calle Juan Esplandiu 11, Madrid, 28007 Spain; 7 Hospital Universitario y Politécnico, La Fe, IIS La Fe. Avinguda de Fernando Abril Martorell, 106, València, 46026 Spain; 8 Centro de Investigacion Biomedica en Red de Enfermedades Raras, ISCIII - Instituto de Salud Carlos III, Avenida Monforte de Lemos, 5, Madrid, 28029 Spain; 9 Department of Medicine, University of Valencia, Avenida de Blasco Ibáñez, 15, València, 46010 Spain

**Keywords:** Spinal muscular atrophy, Patient reported outcomes, Psychometric properties, Quality of life, Treatment outcomes

## Abstract

**Background:**

There is a need to expand the current scope of assessment tools usually applied to patients with Spinal Muscular Atrophy (SMA). This study aimed to assess the psychometric properties (reliability and discriminant validity) of a set of new patient-reported outcome measures (PROMs) called PROfuture, after analysing the performance of individual items of the questionnaires.

**Results:**

Patients included in the Spanish SMA Patient-Reported Registry (RegistrAME) were invited to answer 10 questionnaires: Fatigability; Pain; Scoliosis and Contractures (S&C); Feeding (F); Breathing and Voice (B&V); Sleep and Rest (S&R); Vulnerability; Infections and Hospitalisations (I&H); Time spent in care (T); and Mobility and Independence (M&I). The diagnosis date, type of SMA, functional classification, and comorbidities were also collected. A total of 160 patients of the 330 included in RegistrAME participated in the study: mean age (SD) 18 (16.6) years, 27.5% non-sitter, 46.88% sitter, and 25.63% walker, 20.0% type 1 SMA, 51.88% type 2, and 28.12% type 3. The frequency of symptoms varied from 43.5% of patients reporting some degree of Pain to 96.3% reporting some degree of Fatigability. The reliability assessed by Cronbach’s alpha coefficient was > 0.75 for all the PROs and > 0.9 for S&C, F, B&V, T, and M&I. Regarding content validity, scores were higher (worse health status) in type 1 SMA patients than in types 2 and 3, and were also higher for non-sitter patients than for sitter and walker patients.

**Conclusions:**

The ten questionnaires included in the PROfuture set were developed based on what people living with spinal muscular atrophy and their caregivers consider relevant. This preliminary study provides an initial basis to consider their potential usefulness in assessing aspects that matter to this population. The early findings are promising, however, further extensive psychometric evaluation is needed. PROfuture is a new set of patient-reported outcome measures, specifically designed by and for individuals living with spinal muscular atrophy and their caregivers. Future studies will help strengthen the evidence regarding its reliability and validity.

**Supplementary Information:**

The online version contains supplementary material available at 10.1186/s13023-025-03832-y.

.

## Background

Spinal muscular atrophy (SMA) is a neuromuscular disease characterized by the degeneration of motor neurons in the anterior horn of the spinal cord [1]. The most common cause of SMA is a biallelic mutation in the survival motor neuron 1 (*SMN1*) gene [2, 3]. On average, the estimated combined incidence of all types of SMA is 8 per 100,000 live births, with a prevalence of 1–2 per 100,000 persons [4]. Despite SMA being a rare disease, it has been one of the leading monogenic causes of infant mortality [5]. Moreover, a recently published study has shown that, during the pre-treatment era, patients with SMA of all ages and phenotypes had a higher all-cause mortality rate compared with age-matched controls [6]. The impact on those living with SMA and their families goes beyond the progressive mobility impairment and physical deterioration. SMA patients and their families experience a high level of emotional and social burden due to the loss of functional abilities, expectations, premature death, or social discomfort and stigma, among others [7]. Thus, SMA usually has a huge impact on the health-related quality of life of patients and their families [8–10].

Currently, different motor function scales and questionnaires are used to evaluate the functional state of patients with SMA depending on their phenotype and age. Among them, the Children’s Hospital of Philadelphia Infant Test of Neuromuscular Disorders (CHOP INTEND) [11], the Hammersmith Infant Neurological Examination (HINE) [12], the Hammersmith Functional Motor Scale-Expanded (HFMSE) [13], the Revised Upper Limb Module (RULM) [14], and the Six-minute walk test (6MWT) [15] are the most used scales in Spain.

However, these scales show both floor and ceiling effects, especially in adult patients [16]. Moreover, they focus on motor function, neglecting other areas relevant to patients and that might improve with new treatments. This idea has been underlined in several published studies. One of these [17] highlighted that current scales and questionnaires do not adequately address the full burden of SMA due to the heterogeneity of the disease. The authors stated that it is important to broaden the way these patients are evaluated. Another study [18] provided information about the impact of several symptoms on adult patients with SMA.

Therefore, bearing in mind the need to expand the current scope of assessment tools usually applied to SMA patients, five focus groups with patient experts and caregivers were organised by the Spanish SMA Foundation (FundAME, by its Spanish acronym). The objective of these groups was to obtain information on the impact of the disease on patients’ lives, including physical, psychological, and social aspects, while considering the different phenotypes of the disease. Details on the methodology used and results obtained have been reported elsewhere [19]. As a result, a set of 142 items grouped into 10 domains or questionnaires (PROfuture) were developed. The 10 questionnaires assess different areas: Fatigability, Pain, Scoliosis and Contractures, Feeding, Breathing and Voice, Sleep and Rest, Vulnerability, Infections and Hospitalisations, Time spent in care, and Mobility and Independence. The questionnaire was incorporated as a new module into the FundAME patient registry, RegistrAME, which adheres to a rigorous curation system [20]. All questionnaires were designed with clear and straightforward language to minimise misinterpretation. A pilot phase was conducted to validate the online format and ensure consistency with face-to-face administration [19] (Supplementary material: PROfuture Dataset). To facilitate the patient’s experience and ensure they feel identified with the questions they need to answer, a design based on a computerized assisted administration system was used. This system operates using health data previously entered into the registry and dynamically adapts the questions according to the health data collected, related to variables such as age, motor level, use of a wheelchair, and type of wheelchair (electric, manual, or mixed). The questions are presented specifically and sequentially according to these predefined criteria, ensuring that each participant only sees the relevant questions. This can be seen in the Supplementary Material, where the questions are displayed according to motor level or wheelchair usage.

By doing this, firstly, patients only answer those questionnaires that assess areas of life that they feel impacted and do not receive questions that are out of context and/or emotionally inappropriate, thereby assuring, secondarily, adherence to the response process, in addition to shortening the time that the patient must invest in the task.

In the present study, the performance of individual items and the psychometric properties of the new questionnaires (reliability and discriminant validity) were analysed.

## Methods

A cross-sectional observational study was conducted. Patients included in the Spanish SMA Patient-Reported Registry (RegistrAME) [20] were invited to participate; if the patient was under 12 years old, their parents were invited as surrogates. All patients in the RegistrAME have a confirmed diagnosis of autosomal recessive 5q SMA (genetic confirmation of biallelic mutation predictive of loss-of-function of the *SMN1* gene) and had signed an informed consent to participate in the registry. For patients between 12 and 17 years of age, the assent of the patients and the informed consent of their parents were obtained. For patients between 2 and 12 years old, the parents signed the informed consent. The study protocol was approved by the Ethics Committee of the “Hospital Clínico San Carlos” of Madrid (21/561-E).

. Patients or their parents were asked to answer the set of 10 PROfuture questionnaires via the RegistrAME website. Each questionnaire focuses on a specific domain: Fatigability (19 items), Pain (10 items), Scoliosis and Contractures (12 items), Feeding (21 items), Breathing and Voice (8 items), Sleep and Rest (4 items), Vulnerability (9 items), Infections and Hospitalisations (4 items), Time Spent in Care (10 items), and Mobility and Independence (45 items).

Diagnostic date, SMA type, number of *SMN2* copies, functional classification, and comorbidities were also collected. The data collection started on May 1, 2020, and finished on April 30, 2021.

### Statistical analysis

All analyses were performed using the statistical package SAS^®^.

*Item analysis*. This analysis first sought to verify that the items grouped in each PROfuture questionnaire assessed the same concept, and then to build the score for each questionnaire after deleting items with bad performance. This analysis included (1) the percentage of missing responses for each item; (2) the distribution of responses for each item; and (3) the estimation of item-total correlation coefficients, to assess their discriminative power (homogeneity index). Items with missing values for more than 20% of patients who were expected to respond (some items are only presented based on a response to a previous item), those with a skewed distribution of responses (ceiling or floor effect greater than 20%), and those that showed an item-total correlation coefficient of less than 0.30 were eliminated from the questionnaire. An exploratory factorial analysis was then carried out with the items belonging to each of the questionnaires to group them into dimensions (a questionnaire may have more than one dimension if so determined in this analysis). Subsequently, the score calculation for each questionnaire was performed as a percentage; thus, a higher score indicates a higher impact of the disease.

*Analysis of the domains or questionnaires*. For each scale, the following analyses were carried out: (1) reliability analysis using Cronbach’s alpha coefficient, which should be greater than 0.7; and (2) analysis of the discriminant validity, comparing the scores of the questionnaires among the different groups of patients according to SMA type, age, *SMN2* copy number, and current ambulation status.

## Results

Of the 330 SMA patients enrolled in RegistrAME, 160 agreed to participate in the present study and completed the PROfuture questionnaires. The number of patients who reported an impact on each area and completed the respective questionnaire were: Fatigability (*N* = 155), Sleep and Rest (*N* = 151), Infections and Hospitalisations (*N* = 132), Vulnerability (*N* = 130), Mobility and Independence (*N* = 129), Scoliosis and Contractures (*N* = 121), Breathing and Voice (*N* = 117), Feeding (*N* = 116), Time spent in care (*N* = 115), and Pain (*N* = 70). Only 45 patients reported symptoms in all domains and, consequently, answered all the questionnaires.

According to the type of SMA, 32 patients (20.0%) were classified as type 1, 83 (51.88%) as type 2, and 45 (28.12%) as type 3. For this classification, the onset of symptoms and the maximum motor level achieved were considered. The main characteristics of the sample, according to SMA type, are shown in Table 1. According to functional status, 41 patients were classified as “walkers”, 75 as “sitters” and 44 as “non-sitters”. Among the “walkers”, 18 (43.9%) walked independently and never used a wheelchair, 9 (22%) walked at home alone but needed a wheelchair when leaving home, and 14 (34.1%) walked short routes but used a wheelchair for longer stretches. Among the “sitters”, 4 (5.3%) were able to sit and stand up on their own, 11 (14.5%) were able to sit on their own but not stand, and 61 (80.2%) were not able to sit on their own but could remain seated independently.

**Table 1:** Main sociodemographic and clinical characteristics of the study sample

	SMA 1 (n = 32)	SMA 2 (n = 83)	SMA 3 (n = 45)	TOTAL
**Age. Mean (SD).**	4.56 (4.41)	17.02 (14.07)	29.09 (18.88)	17.93 (16.62)
**Range [min-max]**	[1–21]	[2–62]	[3–76]	[1–76]
**Number of** ***SMN2*** **copies. N (%)**				
** 2 copies**	24 (75.00%)	8 (9.64%)	1 (2.20%)	33 (20.63%)
** 3 copies**	7 (21.88%)	65 (78.31%)	27 (60.00%)	99 (61.88%)
** 4 copies**		3 (3.61%)	16 (35.56%)	19 (11.88%)
**Functional classification. N (%)**				
** Non-sitter**	14 (43.75%)	28 (33.73%)	2 (4.44%)	44 (27.50%)
** Sitter**	15 (46.88%)	49 (59.04%)	11 (24.44%)	75 (46.88%)
** Walker**	3 (9.38%)	6 (7.23%)	32 (71.11%)	41 (25.63%)
**Disease-modifying treatment for SMA. N (%)**				
** Yes**	32 (100%)	60 (72.29%)	33 (73.33%)	125 (78.13%)
** No**	-	23 (27.71%)	12 (26.67%)	35 (21.87%)

To account for differences in responses from patients and proxies, we identified who was responding to the questionnaire in the registry. All responses for participants under the age of 15 were completed by parents, while participants aged 21 or older completed the questionnaire independently. For participants aged 16 to 20, responses were mixed between patients and parents. While all responses were analysed collectively in this study due to sample size limitations, future research will separately analyse patient and proxy data to explore potential differences.

### Item analysis

Items with bad performance according to pre-specified criteria were eliminated from the questionnaire. The following items were eliminated: Feeding (five items referring to gastric button feeding were removed for scoring purposes due to a low item-total correlation coefficient, although they will be kept in the questionnaire for descriptive purposes for a specific group of the SMA patient population), Breathing and Voice (one item was deleted because a high number of values were missing), Vulnerability (two items were removed because of a high ceiling effect), Infections and Hospitalisations (a score could not be generated for this dimension, although it was considered useful for descriptive purposes), and Mobility and Independence (seven items removed due to a ceiling effect, eight items due to a floor effect, and one additional item due to a low item-total correlation coefficient). No items were removed from Fatigability, Pain, Scoliosis and Contractures, Sleep and Rest, and Time spent in care.

Exploratory factor analysis allowed us to identify different sub-dimensions in most of the questionnaires (Fatigability-2 factors; Pain-5 factors; Scoliosis and Contractures-3 factors; Feeding-2 factors; Breathing and Voice-2 factors; Vulnerability-4 factors; Time spent in care-2 factors; and Mobility and Independence-3 factors), which accounted for more than 80% of the score variance. Despite this, the research team decided to build only one global score for each questionnaire, considering that the first loading factor in each questionnaire accounted for ≥ 50% of the score variance and that item-total correlation was high in all cases. A score was not calculated for Sleep and Rest because it has one factor for every single item of the questionnaire, nor for Infections and Hospitalisations as it was decided to use them only for descriptive purposes (see above).

### Analysis of the questionnaires

**Fig. 1:**
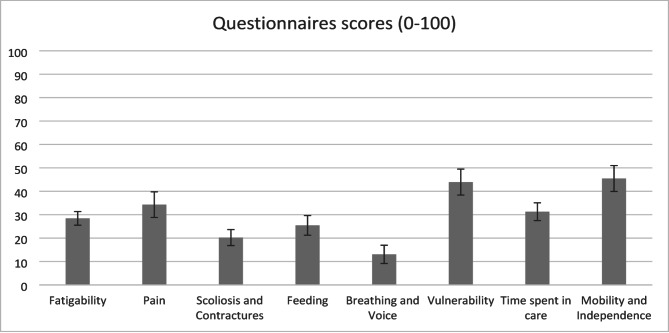
Mean scores and standard error of the PROfuture questionnaires

The mean total scores of the questionnaires, after deleting items with bad performance, are presented in Fig. 1. For Sleep and Rest and Hospitalisations, where a total score was not calculated, the answer distribution is presented in Table 2.

**Table 2:** Distribution of items of sleep and rest and hospitalisations

Sleep and Rest items		Total	Hospitalisations items		Total
1. Have you woken up during the night to ask for help and to be helped to move in bed?	Valid N	151	1. Number of respiratory infections during the last month	Valid N	132
	Never	47 (31.13%)		0	119 (90.15%)
	Rarely	17 (11.26%)		1	6 (4.55%)
	Sometimes	20 (13.25%)		Missing	7 (5.30%)
	Very often	16 (10.60%)	2. Number of visits to the emergency room in the last 3 months	Valid N	132
	Always	48 (31.79%)		0	110 (83.33%)
	Missing	3 (1.99%%)		1	14 (10.61%)
1a. The nights that you have woken up, how many times is usual?	Valid N	95		2	1 (0.76%)
	1 time	26 (27.37%)		Missing	7 (5.30%)
	2–3 times	49 (51.58%)	3. Number of days admitted to the hospital or at home during the last 3 months	Valid N	132
	4 + times	20 (21.05%)		0	115 (87.12%)
2. If you use a ventilation device, did you wake up during the night because it bothered you?	Valid N	151		1	4 (3.03%)
	Never	59 (39.07%)		2	3 (2.27%)
	Rarely	16 (10.60%)		7+	1 (0.76%)
	Sometimes	20 (13.25%)		Missing	9 (6.82%)
	Very often	2 (1.32%)	3a. Of the days in the hospital, how many were in the intensive care unit during the last 3 months?	Valid N	8
	Always	6 (3.97%)		0	8 (100%)
	Missing	48 (31.79%)			
3. Have you woke up tired after sleeping at night?	Valid N	151			
	Never	45 (29.80%)			
	Rarely	53 (35.10%)			
	Sometimes	35 (23.18%)			
	Very often	7 (4.64%)			
	Always	1 (0.66%)			
	Missing	56 (37.09%)			

To assess the internal consistency of each domain, Cronbach’s alpha coefficients were calculated for the total scores of all ten PROfuture questionnaires. As shown in Table 3, all scales demonstrated good to excellent internal reliability, with alpha values ranging from 0.78 to 0.97.

**Table 3:** Internal consistency (Cronbach’s alpha) of profuture questionnaires

Questionnaire	Cronbach’s alpha
Fatigability	0.83
Pain	0.78
Scoliosis and Contractures	0.91
Feeding	0.97
Breathing and Voice	0.95
Vulnerability	0.78
Time Spent in Care	0.94
Mobility and Independence	0.97

Differences in questionnaire scores between different groups of patients based on the type of SMA and current ambulation status are presented in Tables 4 and 5 respectively; and those according to age and the number of *SMN2* copies are presented in Tables 6 and 7. Overall, the questionnaires discriminated well between the different subgroups of patients.

**Table 4:** Scores of the profuture questionnaires according to SMA type classification

Variable		SMA 1	SMA 2	SMA 3	Total
Scores (0–100) for Fatigability	Valid N	31	81	42	155
	Mean (SD)	30.38 (24.71)	28.97 (16.78)	26.25 (15.18)	28.43 (18.16)
Scores (0–100) for Pain	Valid N	10	36	19	65
	Mean (SD)	20.01 (19.39)	37.46 (21.91)	35.83 (21.80)	34.30 (22.07)
Scores (0–100) for Scoliosis and Contractures	Valid N	26	63	25	115
	Mean (SD)	19.56 (15.87)	23.55 (20.71)	13.38 (11.39)	20.23 (18.33)
Scores (0–100) for Feeding	Valid N	23	63	23	110
	Mean (SD)	40.51 (26.28)	24.69 (19.93)	13.29 (13.62)	25.44 (22.04)
Scores (0–100) for Breathing and Voice	Valid N	25	63	23	112
	Mean (SD)	26.36 (24.34)	11.48 (13.25)	3.48 (6.30)	13.06 (17.20)
Scores (0–100) for Vulnerability	Valid N	25	72	28	126
	Mean (SD)	47.68 (27.79)	44.36 (25.96)	40.26 (28.91)	43.92 (26.87)
Scores (0–100) for Time spent in care	Valid N	16	63	29	109
	Mean (SD)	41.03 (16.95)	30.99 (16.37)	27.33 (16.19)	31.28 (16.89)
Scores (0–100) for Mobility and Independence	Valid N	19	65	29	114
	Mean (SD)	63.41 (27.02)	50.62 (20.68)	23.69 (20.04)	45.47 (25.81)

**Table 5:** Scores of the profuture questionnaires according to current ambulation status

Variable		Non-sitter	Sitter	Walker	Total
Scores (0–100) for Fatigability	Valid N	42	72	41	155
	Mean (SD)	41.81 (21.35)	21.59 (12.73)	26.74 (15.46)	28.43 (18.16)
Scores (0–100) for Pain	Valid N	19	29	17	65
	Mean (SD)	41.24 (22.82)	31.05 (20.21)	32.08 (23.81)	34.30 (22.07)
Scores (0–100) for Scoliosis and Contractures	Valid N	33	58	24	115
	Mean (SD)	30.65 (21.94)	18.81 (15.39)	9.34 (11.10)	20.23 (18.33)
Scores (0–100) for Feeding	Valid N	31	56	23	110
	Mean (SD)	36.32 (28.08)	24.05 (18.06)	14.19 (14.54)	25.44 (22.04)
Scores (0–100) for Breathing and Voice	Valid N	33	56	23	112
	Mean (SD)	19.58 (16.68)	10.87 (13.99)	9.02 (22.49)	13.06 (17.20)
Scores (0–100) for Vulnerability	Valid N	35	65	26	126
	Mean (SD)	56.82 (23.89)	39.56 (26.40)	37.44 (26.91)	43.92 (26.87)
Scores (0–100) for Time spent in care	Valid N	29	56	24	109
	Mean (SD)	32.21 (18.24)	33.14 (16.53)	25.81 (15.52)	31.28 (16.89)
Scores (0–100) for Mobility and Independence	Valid N	33	57	24	114
	Mean (SD)	69.72 (16.71)	44.03 (18.83)	15.56 (15.46)	45.47 (25.81)

**Table 6:** Scores of the profuture questionnaires according to age

Variable		< 2 years	2–6 years	7–10 years	11–15 years	> 15 years	Total
Scores (0–100) for Fatigability	Valid N	2	46	22	18	67	155
	Mean (SD)	31.25 (8.84)	25.73 (18.36)	27.46 (19.36)	21.82 (9.87)	32.30 (19.01)	28.43 (18.16)
Scores (0–100) for Pain	Valid N	0	13	6	10	36	65
	Mean (SD)	. (.)	17.92 (16.12)	30.85 (13.12)	34.72 (18.35)	40.67 (23.39)	34.30 (22.07)
Scores (0–100) for Scoliosis and Contractures	Valid N	2	36	14	10	53	115
	Mean (SD)	30.80 (0.00)	12.59 (13.16)	16.88 (14.34)	26.69 (18.53)	24.69 (20.80)	20.23 (18.33)
Scores (0–100) for Feeding	Valid N	1	35	12	10	52	110
	Mean (SD)	50.80 (.)	26.39 (18.41)	26.13 (24.08)	16.09 (5.13)	25.96 (25.52)	25.44 (22.04)
Scores (0–100) for Breathing and Voice	Valid N	2	36	13	10	51	112
	Mean (SD)	37.50 (53.03)	16.44 (20.68)	4.00 (4.90)	7.20 (9.00)	13.16 (14.84)	13.06 (17.20)
Scores (0–100) for Vulnerability	Valid N	2	36	18	14	56	126
	Mean (SD)	42.00 (59.40)	39.01 (24.17)	29.19 (24.82)	43.06 (20.30)	52.09 (27.61)	43.92 (26.87)
Scores (0–100) for Time spent in care	Valid N	1	24	17	13	54	109
	Mean (SD)	26.30 (.)	35.00 (18.62)	31.47 (15.78)	42.82 (17.80)	26.88 (14.99)	31.28 (16.89)
Scores (0–100) for Mobility and Independence	Valid N	1	27	17	13	56	114
	Mean (SD)	25.70 (.)	51.56 (28.72)	37.65 (24.75)	45.75 (16.26)	45.20 (26.43)	45.47 (25.81)

**Table 7:** Scores of the profuture questionnaires according to *SMN2* copy number

Variable		2 copies	3 copies	4 copies	Total
Scores (0–100) for Fatigability	Valid N	31	95	20	148
	Mean (SD)	26.55 (21.93)	30.31 (16.97)	23.76 (19.23)	28.61 (18.35)
Scores (0–100) for Pain	Valid N	10	36	19	65
	Mean (SD)	20.01 (19.39)	37.46 (21.91)	35.83 (21.80)	34.30 (22.07)
Scores (0–100) for Scoliosis and Contractures	Valid N	26	71	14	112
	Mean (SD)	16.77 (12.46)	22.51 (19.89)	15.00 (19.30)	20.09 (18.42)
Scores (0–100) for Feeding	Valid N	24	68	14	107
	Mean (SD)	33.01 (22.13)	26.10 (22.43)	12.24 (16.37)	25.65 (22.29)
Scores (0–100) for Breathing and Voice	Valid N	25	69	14	109
	Mean (SD)	24.25 (24.49)	10.89 (13.47)	2.86 (6.55)	12.86 (17.29)
Scores (0–100) for Vulnerability	Valid N	26	79	16	122
	Mean (SD)	44.98 (29.77)	41.54 (24.81)	45.09 (30.34)	43.05 (26.61)
Scores (0–100) for Time spent in care	Valid N	18	70	16	105
	Mean (SD)	40.25 (13.74)	30.45 (17.26)	25.51 (17.32)	31.27 (17.11)
Scores (0–100) for Mobility and Independence	Valid N	22	71	16	110
	Mean (SD)	52.59 (29.67)	46.39 (23.27)	27.02 (26.91)	44.92 (26.06)

## Discussion

The motor scales and questionnaires currently used in patients with SMA do not usually assess the full burden of this disease [17, 18]. Therefore, there is a need for new measures incorporating other important areas of patients’ lives that are not covered by the pre-existing tools [21, 22]. Thus, to expand the scope of the assessment tools currently applied to SMA patients, ten new questionnaires (PROfuture) were developed by a multidisciplinary team of SMA patients and experts. A previous multicentre study assessed four of these questionnaires (Fatigability, Breathing and Voice, Sleep and Rest, and Vulnerability) along with other outcome measurements (SMA Tool) in a multicentre clinical-based setting [23]. In contrast, in the present study, all ten questionnaires were assessed online in a home-based setting. After analysing individual item functioning and deleting some items with bad performance according to prefixed criteria, total scores were built for each questionnaire, except for Sleep and Rest and Infections and Hospitalisations. The questionnaires showed good reliability and discriminant validity.

The exploratory factor analysis showed multidimensionality in all questionnaires analysed, nevertheless, the research group decided to build only one total score for each questionnaire in order to facilitate the interpretability of the scores when using the questionnaires in routine clinical practice. Total scores were not created for Sleep and Rest and Infections and Hospitalisations, however, the small number of items in these questionnaires makes them useful to assess their respective areas, without the need for a total score.

All questionnaires but one (Pain) were answered by more than 70% of patients. Fatigability was the most frequently reported area of impairment (97% of patients). This was followed by Vulnerability, Scoliosis and Contractures, and Mobility and Independence, regardless of the SMA type or functional subgroup This discrepancy in participation suggests a potential influence of the program’s design. Originally, the program was conceived for use on laptops or tablets and was not fully adapted for mobile devices. This lack of adaptation could have restricted the visibility of certain areas on mobile devices, thereby potentially affecting participants’ engagement in those areas.

The highest scores were observed in Vulnerability, Mobility and Independence, Pain, and Time spent in care, indicating that some areas that were not so frequently reported may also have a significant impact on patients’ lives. Some areas, like Mobility and Independence and Pain, are assessed in other questionnaires, such as the SMAIS [24], the EK2 scale [25], and SMA-HI [26]; however, it is remarkable that other areas, like Vulnerability and Time spent, in care are not explored by other tools. This is even more important in the case of Vulnerability, which was answered by 81% of participants and showed the highest impact of all questionnaires. It is worth noting that the Pain questionnaire was answered by less than half of the patients, yet its total score was the third highest of the ten questionnaires. This suggests that, although pain is less frequent than other symptoms, it may have a significant impact on some SMA patients. The mean scores (standard deviation) for Fatigability, Breathing and Voice, and Vulnerability obtained by the patients in the previous study using these questionnaires [23] were 21.86 (19.66), 8.88 (15.18,) and 13.41 (18.36), respectively. These scores were slightly lower than those of the present study for Fatigability and Breathing and Voice, and considerably lower in the case of Vulnerability. However, the sample included herein incorporated more patients under eight years of age than the previous study (31.25% vs. 24.77%) and more patients with more severe forms of the disease (SMA 1 patients: 20% vs. 7.96%).

The Fatigability questionnaire confirmed previous results [23] of good reliability, with a Cronbach’s alpha close to 0.8, and good discriminant validity, showing higher scores in patients with younger onset (type 1) and worse function (non-sitter). A greater impact of fatigability on non-sitter patients had been previously suggested in a preliminary study, using the same questionnaire, but focusing on adolescent and adult patients [27].

The Pain questionnaire showed good reliability, like Fatigability. The discriminant validity was also good for ambulation status (it was higher in non-sitters than in sitters vs. walkers), but not for SMA type: Pain scores were higher in patients with SMA types 2 and 3, compared with type 1. Moreover, there was a consistent increase in Pain scores with age (Table 6), suggesting that pain is less frequent and severe in children under 11 years old.

Scoliosis and Contractures showed very good reliability, with a Cronbach’s alpha > 0.9. Concerning discriminant validity, scores of non-sitter patients were higher than those of sitter and walker patients, and scores of type 2 patients showed the highest impact of this area on this group of patients compared with type 1 and type 3 patients. Again, this suggests an increasing impact of scoliosis and contractures with age in non-sitter patients. Consistently, there is an increase in scores with age (Table 6), particularly between 11 and 15 years old, reflecting the natural history of the disease [28].

The Feeding questionnaire demonstrated very good reliability (Cronbach’s alpha = 0.97) and good discriminant validity, presenting higher scores in patients with more severe disease.

Cronbach’s alpha of the Breathing and Voice questionnaire was 0.95, showing very good reliability. Discriminant validity was also good since patients with more severe disease had higher scores, as found in the previous study [23].

The Vulnerability questionnaire showed good reliability and discriminant validity, as in the previous study [23].

The Time spent in care questionnaire showed very good reliability (Cronbach’s alpha = 0.94). The discriminant validity was good for SMA type and severity but, interestingly, scores were similar for non-sitter and sitter patients, suggesting that ambulation is key for determining the time spent in care.

Mobility and Independence showed a very high Cronbach’s alpha coefficient, indicating very good reliability. Discriminant validity was also good as patients with more severe disease had higher scores.

PROFuture PROM comprises a set of 10 different questionnaires that specifically assess the areas of life that are more relevant to a specific patient in order to detect small changes that could be meaningful for that subject. The research team considered this mode of assessing patients’ symptoms more useful (instead of developing a unique score by adding the scores from all dimensions), and probably more sensitive to changes, for the evaluation of future treatments.

### Limitations of the study

One limitation of the present study is that data was collected through the website of the Fundame registry, which may skew the profile of patients evaluated towards those more engaged. A second limitation is that the sample includes very heterogeneous patients regarding demographic and disease-related characteristics, combining both patients’ and parents’ responses, which might make the interpretation of the results difficult. However, the objective of the study was precisely to provide an overview of disease burden over the whole disease spectrum and, in children under 12 years old, parents are the most reliable informants. Moreover, despite this limitation, we were able to demonstrate the good or very good reliability and validity of most questionnaires.

While all responses were analysed collectively in this study due to sample size limitations, identifying patient and proxy responses allowed us to ensure clarity in the data. Future research will build on this foundation by separately analysing these groups to further refine the tool and enhance its applicability across diverse respondent types.

The data were collected from a self-reported patient registry, which inherently relies on the proactivity and willingness of patients to participate. This may influence the profile of the evaluated patients, as those who are more engaged or proactive could be overrepresented, potentially leading to a less diverse sample. The criterion validity could not be assessed as no other questionnaire (RULM, HFMSE…) was collected from participants in this study. Additionally, convergent and criterion validity will be established to further validate PROfuture. Plans are already in place to evaluate its correlations with established clinical tools (e.g., RULM, HFMSE) and to explore its relationship with broader clinical endpoints.

## Conclusions

The PROfuture questionnaires, developed by patients for patients in the current era of new treatments for SMA, can help to better characterize the impact of SMA on different areas of patients’ lives that are currently unexplored by other tools. These questionnaires can be implemented both in home- and clinical-based settings. Moreover, they can be used together (as a comprehensive tool) or separately, depending on the objectives of the study, making them ideal for their use in research settings. Future longitudinal studies should assess the sensitivity to change of the PROfuture questionnaires in a home-based setting so that they can be incorporated into clinical trials and practice.

Additionally, convergent and criterion validity will be established to further validate PROfuture. Plans are already in place to evaluate its correlations with established clinical tools (e.g., RULM, HFMSE) and to explore its relationship with broader clinical endpoints. This step will be essential to position PROfuture as a reliable and robust outcome measure for both clinical and research applications.

## Electronic supplementary material

Below is the link to the electronic supplementary material.

Supplementary Material 1

## Data Availability

The datasets used and analysed during the current study are not publicly available. This is to protect the study participants’ privacy. Data could be available from the corresponding author upon reasonable request.

## Abbreviations

SMA: Spinal Muscular Atrophy
PROMs: Patient-reported outcome measures
S&C: Scoliosis and Contractures
F: Feeding
B&V: Breathing and Voice
S&R: Sleep and Rest
I&H: Infections and Hospitalisations
T: Time spent in care
M&I: Mobility and Independence
SMN1: Survival motor neuron 1 gene
CHOP INTEND: Children’s Hospital of Philadelphia Infant Test of Neuromuscular Disorders
HINE: Hammersmith Infant Neurological Examination
HFMSE: Hammersmith Functional Motor Scale-Expanded
RULM: Revised Upper Limb Module
6MWT: Six-minute walk test
